# Cost and Affordability of Diets Modelled on Current Eating Patterns and on Dietary Guidelines, for New Zealand Total Population, Māori and Pacific Households

**DOI:** 10.3390/ijerph15061255

**Published:** 2018-06-13

**Authors:** Sally Mackay, Tina Buch, Stefanie Vandevijvere, Rawinia Goodwin, Erina Korohina, Mafi Funaki-Tahifote, Amanda Lee, Boyd Swinburn

**Affiliations:** 1 School of Population Health, University of Auckland, Auckland 1142, New Zealand; s.vandevijvere@auckland.ac.nz (S.V.); rawiniagoodwin@icloud.com (R.G.); boyd.swinburn@auckland.ac.nz (B.S.); 2 The Heart Foundation of New Zealand, Auckland 1051, New Zealand; TinaB@heartfoundation.org.nz (T.B.); mafift@heartfoundation.org.nz (M.F.-T.); 3 Toi Tangata, Auckland 1010, New Zealand; erina@toitangata.co.nz; 4 The Australian Prevention Partnership Centre, The Sax Institute, Sydney 1240, Australia; amanda.lee@saxinstitute.org.au

**Keywords:** INFORMAS, diet prices, food affordability, Pacific diets, Māori diets, food security

## Abstract

The affordability of diets modelled on the current (less healthy) diet compared to a healthy diet based on Dietary Guidelines was calculated for population groups in New Zealand. Diets using common foods were developed for a household of four for the total population, Māori and Pacific groups. Māori and Pacific nutrition expert panels ensured the diets were appropriate. Each current (less healthy) diet was based on eating patterns identified from national nutrition surveys. Food prices were collected from retail outlets. Only the current diets contained alcohol, takeaways and discretionary foods. The modelled healthy diet was cheaper than the current diet for the total population (3.5% difference) and Pacific households (4.5% difference) and similar in cost for Māori households (0.57% difference). When the diets were equivalent in energy, the healthy diet was more expensive than the current diet for all population groups (by 8.5% to 15.6%). For households on the minimum wage, the diets required 27% to 34% of household income, and if receiving income support, required 41–52% of household income. Expert panels were invaluable in guiding the process for specific populations. Both the modelled healthy and current diets are unaffordable for some households as a considerable portion of income was required to purchase either diet. Policies are required to improve food security by lowering the cost of healthy food or improving household income.

## 1. Introduction

Dietary risks and a high body mass index are major risk factors contributing to health loss globally and in New Zealand (NZ) with dietary risk factors contributing to the highest proportion of total disability-adjusted life years in 2015 compared to other risk factors [1]. New Zealanders consume too much saturated fat, sodium and sugar and not enough dietary fibre, fruit and vegetables [2]. NZ has high rates of obesity with 32.2% of all adults, 50.2% of Māori adults and 68.7% of Pacific adults, obese [3]. For children (aged 2 to 14), 11% of the total population, 18.1% of Māori and 29.1% of Pacific children are obese [3]. Māori and Pacific people are more likely than non-Māori and non-Pacific to experience food insecurity [2].

An ‘obesogenic’ environment is ‘the sum of influences that the surroundings, opportunities, or conditions of life have on promoting obesity in individuals or populations’ [4]. A focus on creating healthy food environments is required to move populations towards diets that meet food-based dietary guidelines [5]. It is fundamental to consider cultural factors when discussing environmental influences on obesity [6].

Non-Māori are more advantaged than Māori across socioeconomic indictors related to education, employment, income and household crowding [7]. Inequities in health outcomes for Māori are influenced by the negative experiences of colonisation, institutional racism, alienation of land and thus identity and historical trauma [8]. In NZ, the Pacific Island community is a large and diverse ethnic group. Pacific communities, while being an integral part of New Zealand’s society, continue to face challenges with lower levels of education and qualifications, lower incomes and a higher unemployment rate than the total population [9].

The International Network for Food and Obesity/NCDs Research, Monitoring and Action Support (INFORMAS) aims to monitor key aspects of food environments related to obesity and non-communicable diseases (NCDs) [10]. The INFORMAS food price module provides a framework to examine the price differential of healthy and unhealthy foods, meals and diets with this research focusing on the diet component.

Food prices are a major influence on household food purchases [11]. When the household budget is limited, fixed costs are prioritized so the money allocated for food reduces, which often results in food insecurity with potential health consequences [12].

Researchers have successfully used expert or focus panels to develop diets and select pricing outlets to ensure the costing of diets reflects intakes [13,14]. This is important in this research as eating patterns of Māori and Pacific households in NZ are influenced by traditional foods and eating patterns.

The relative difference in the affordability of a diet modelled to meet dietary guidelines compared with a modelled current (less healthy) diet has not been measured before in NZ, and there are few international studies. A systematic review by Rao et. al. (2013) concluded that healthier diets cost more than less healthy diets, though this depended on whether the cost of the total diet or cost per 2000 kcal was compared [15].

The affordability of a healthy diet compared to the current diet can be used to estimate the affordability component of food security for households on different income levels, for social planning and to advocate for fiscal policies and examine the influence on diet cost of taxes and subsidies on foods [16,17].

This study aims to assess the affordability of diets modelled on current eating patterns (current diet) and on dietary guidelines (healthy diet), for the total population, Māori and Pacific households, and to explore the feasibility of using expert panels to guide the process.

## 2. Materials and Methods

**Figure 1:**
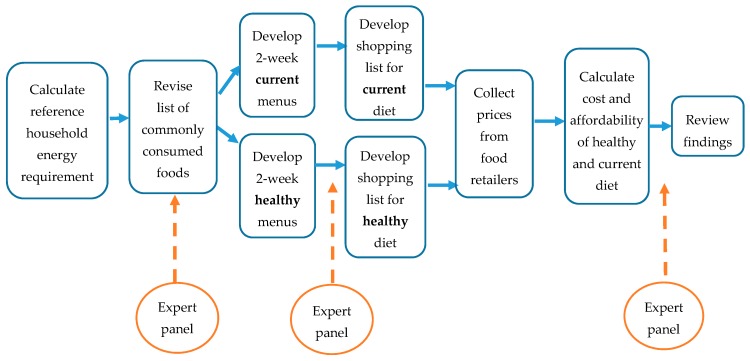
Phases in assessing the cost of a healthy and the current diet.

The methodology follows the guidelines set out in the INFORMAS food prices foundation paper [10] and the INFORMAS food prices module (www.INFORMAS.org). Māori and Pacific expert panels provided guidance for the selection of common foods, menus and price collection methods appropriate to the population group. Figure 1 illustrates the phases in assessing the cost of a modelled healthy versus the current diet. The diets for the total population were developed by a Registered Nutritionist (SM) rather than an expert panel.

The research was approved by the University of Auckland Human Participant Ethics Committee on 22 June 2016 for the Pacific diets (reference 017579) and on 26 September 2016 for the Māori diets (reference 018028). All expert panel participants provided written informed consent prior to participation.

### 2.1. Expert Panels

The members of the Māori (four members) and Pacific (six members) expert panels were health professionals knowledgeable about foods and dietary patterns of their communities. The members were selected on the advice of Māori and Pacific non-governmental health organisations.

Phase 1: The expert panel reviewed a list of commonly consumed foods for Māori or Pacific people, provided feedback on menus for the diets and suggested the type and location of retailers for price collection. The initial discussion was face-to-face. The revised commonly consumed foods list and menu plans were emailed to the experts for review.

Phase 2: The results were presented to each expert panel who provided input into the interpretations and implications of the findings.

### 2.2. Common Foods

Commonly consumed foods were identified from the micro-data of the 2008/09 Adult Nutrition Survey [18] for the total population and for Māori and Pacific separately. Within each of the major food groups (33), the minor groups (395) with the most people consuming the item, or the most grams consumed were identified. Foods consumed by at least 5% of people were considered frequent. The amount consumed of a minor group depended on the food, for example, bread was consumed in higher amounts than butter.

A list of 109/107 common foods was presented to the Māori and the Pacific expert panels respectively. Items were then added or excluded based on the consensus of the expert panel on whether the foods were frequently consumed by the respective population group [Table S1]. The revised Māori common food list included traditional foods such as watercress and mussels. The revised Pacific common food list included taro, green bananas, cabin bread, canned corned beef, mutton flaps, panipopo (sweet coconut buns) and coconut cream.

The number of foods included on the list needed to be manageable for price collection, while ensuring sufficient variety for a two-week menu. The initial revised lists were too extensive so were refined by expert consensus, with some foods acting as proxies for similar foods e.g., jam represented all sweet spreads. The final selection contained 106 foods for the total population, 120 foods for Māori and 127 foods for Pacific populations.

### 2.3. Household Energy Requirements

The reference household used was that recommended in the INFORMAS food prices module: 45-year old man, 45-year old woman, 14-year old boy, 7-year old girl. The energy requirement for the adults for the healthy diet was calculated using the Body Weight Planner [19] based on a weight derived from a Body Mass Index (BMI) of 23 kg/m^2^ calculated from mean population height [20] for moderate physical activity [Table S2]. The energy requirement for children for the healthy diet was based on the recommended energy requirements per KJ/kg per day by FAO/WHO/UNU [21] for moderate physical activity. The target weight was calculated from the 50th percentile BMI from the Centres for Disease Control and Prevention growth charts [22] using mean height [20].

The energy requirement for the current diet for adults was based on the current BMI [Table S2]. The average physical activity level (PAL) was unknown for the population, but approximately half of NZ adults met the physical activity guidelines [20] so a moderate physical activity level was selected. The energy requirement for the current diet for children was based on actual weight [20] and moderate physical activity as most children met the NZ physical activity guidelines [23]. The additional energy required for the actual weight was calculated using a validated equation for the excess energy intake per unit excess weight in childhood [24].

### 2.4. Diet Constraints

The current diets were modelled to reflect the median intake of the macronutrients (percentage of energy), fibre and total sugar, serves of fruits, vegetables, grains, meat and alternatives and dairy products reported in the 2008/09 Adult Nutrition Survey [2] and the Children’s Nutrition Survey 2002 [25] [Tables S3 and S4]. The estimated intakes of sodium were from a later survey using a 24-h urine collection [26]. The current diets met Recommended Dietary Intakes (RDIs) and Adequate Intakes (AIs) for micronutrients except for iodine for all household members, and calcium, iron or Vitamin A for some household members.

The healthy diets were modelled to meet the NZ Eating and Activity Guidelines food group recommendations for number of servings [27] [Table S3] and the acceptable macronutrient distribution range, adequate intake for fibre and the upper limit for sodium (2.3 g per day) from the Nutrient Reference Values and the RDIs and AIs for micronutrients [28] [Table S4]. The intake of iodine could not be assessed due to incomplete food composition data on this micronutrient. The RDI for iron was not met by the adult female of each population group. Foods recommended by the Eating and Activity Guidelines (for example, whole-grain bread, lean meat, reduced-fat milk) were selected. There were no discretionary foods (high in added salt, sugar, saturated fat) in the standard healthy diets. An additional scenario was modelled which replaced 6% of energy from a wide range of foods with discretionary foods and alcohol (adult’s diets) to compare a realistic healthy diet rather than an aspirational diet with the current diet.

Additional healthy foods were added to the list of common foods to enable the NZ Eating and Activity Guidelines recommendations for whole-grains, low-fat dairy and legumes to be met; for example, unsalted peanuts, reduced fat corned beef, brown rice, hummus, canned beans. The additional foods were selected based on frequency of consumption in the nutrition surveys and advice from the expert panels.

### 2.5. Gifting and Gathering of Food

The Māori expert panel identified the gifting and gathering of kai (food) as an important part of accessing food. Foods commonly gifted and/or gathered were seasonal fruit and vegetables and seafood. An additional scenario was analysed where the foods commonly gifted or gathered were priced in the original diet at $0 (mandarins, fresh fish, mussels, puha and watercress).

### 2.6. Menu Development

A fortnightly menu was developed for the current and healthy diets for each household member separately using the commonly consumed foods for breakfast, lunch, dinner, snacks and beverages. The expert panels advised on the menu structure. For example, the Pacific expert panel highlighted that on Sunday there is a large shared church feast so people only have a cup of tea and cabin bread for breakfast. The Māori expert panel considered it important to include sauces and spreads in the healthy diet to ensure the diet was realistic. The nutrient content of the menus was analysed using FoodWorks [29] with the NZ Food Composition Database. The nutrient composition of some Pacific foods were entered as additional foods, sourced from the Pacific Island Food Composition Tables [30]. Modifications were made to ensure diets met the constraints.

### 2.7. Price Collection

The amount to purchase for the household, allowing for inedible portion, yield and retention factors, [31] was calculated [Tables S5 and S6]. The expert groups advised that households would select the cheapest brand. Therefore, the brand with the cheapest price was collected from each store, including discount prices and generic brands. For items sold per unit, for example head of broccoli or a donut, three units were weighed and averaged to calculate the price per 100 g.

For the total population, prices were collected from a convenience sample of twelve supermarkets representing the three major supermarket chains and twelve neighbouring fresh produce stores in greater Auckland in November 2016 over two weeks. The prices for takeaway items were sourced from the INFORMAS meals cost study [32]. All items were available.

The Māori expert panel advised to collect prices from urban and rural grocery stores because price and access may be a barrier in rural areas. Prices were collected from three supermarkets (two large, one small) in an urban area and from three supermarkets (one large, two small) in rural areas and takeaway outlets in the Waikato region. Price collection was for one week in July 2017. Six items were not available in some of the smaller grocery stores, mainly fresh fish and meat.

The Pacific expert panel advised that prices should be collected in South Auckland to ensure specific Pacific foods were available. Prices were collected from three supermarkets (each major chain), three neighbouring fruit and vegetable shops, three bakeries and takeaway outlets. Price collection was for two weeks in September 2016. Not all items were available in stores such as mutton flaps, wholemeal pasta, light coconut cream and taro leaves.

### 2.8. Analysis

The cost of the household diet was calculated for the healthy and current diet (Table 1) for the three populations. A scenario was calculated with the 15% Goods and Services Tax (GST) removed from core foods (fruits, vegetables, less processed meat, seafood, poultry, legumes, nuts, dairy, healthy oils, grains).

To assess affordability of the diets, the percentage of household income required to purchase each diet was calculated for three scenarios:

Scenario 1: Median disposable income [33]

Scenario 2: Household receiving income support

- Jobseeker Support [34]
- Accommodation Supplement (area 2) [34]
- Family tax credit [35]

Scenario 3: Minimum wage [36]

- 60 h per week = one adult 40 h + one adult 20 h Jobseeker Support [34]
- Family tax credit calculated online using gross wages [35]

## 3. Results

### 3.1. Energy Requirements

The household energy requirement for the modelled healthy diet is 39.9 MJ and for the current diet is 43.6 MJ for the total population, 46 MJ for Māori and 47.3 MJ for Pacific. The current diet has 8.5% more energy than the healthy diet for the total population household, 13.3% for Māori and 15.6% for Pacific households.

### 3.2. Cost of Diets

The cost of the diets, and composite food groups, for each population group is outlined in Table 1. For the total population and Pacific Island households, the cost of a modelled healthy diet per fortnight is slightly less than the current diet by 3.5% and 4.5%, with a cost differential over one year of $588 and $575 respectively. For the Māori household, the cost of a healthy and current diet is similar (0.57% difference). When the diets are equivalent in energy, the healthy diet is more expensive than the current diet for all population groups (by 8.5% for the total population, 13.3% for Māori, and 15.6% for Pacific). When 6% of energy in the healthy diet is replaced by discretionary foods and alcohol, the healthy diet reduces in cost by 0.5% ($3.23 per fortnight).

Discretionary foods, beverages and takeaways comprise 36%, 46% and 41% respectively of the current diet costs for the total population, Māori and Pacific Islander populations. The healthy diets have more protein foods, vegetables, grains, fruit and dairy foods than the current diets, and no takeaways, discretionary foods, alcohol, or sugary beverages.

### 3.3. Affordability of Diets

The percentage of income required to purchase either diet is outlined in Table 2. When the 15% Goods and Services Tax (GST) is removed from core foods, affordability for a household improves more for the healthy diet than the current diet.

### 3.4. Cost Scenarios

For Māori, six items were identified as foods typically gathered or gifted rather than purchased. The modelled healthy diet reduces in cost more than the current diet when these foods are gifted, as all these foods were healthy. In rural areas, the healthy diet cost reduces by $28.34 per week and the current diet cost reduces by $15.00 while in urban areas these figures were $27.23 and $14.20 respectively. Both the healthy and current diets are cheaper in the urban area compared to the rural area, with the healthy diet costing 9.4% more and the current diet 7.6% more in rural areas.

## 4. Discussion

This study showed that in NZ, a diet modelled on dietary guidelines is not more expensive than the current, less healthy diet, however when the diets are equivalent in energy the healthy diet is more expensive than the current diet for all population groups. For the total population and Pacific, the cost of a healthy diet is slightly cheaper than the current, less healthy diet. The current diets are higher in energy than the healthy diets because household energy requirement is determined by the average current BMI for the current diet, which is higher than the BMI used for the healthy diet to maintain weight at a healthy BMI.

The input from the Māori and Pacific expert panels was invaluable to identify some popular foods and practices, the type of food to price, meal patterns, common type of retailers and the importance of gathered and gifted food.

An Australian pilot study using similar methodology found the modelled healthy diet cost approximately 12% less than the modelled current diet for a household of four [37]. The healthy diet had 9.6% less energy than the current diet. The energy requirement of the healthy diet was that required to maintain the current BMI and physical activity level of the population. In the New Zealand study, the energy requirement of the healthy diet was determined by a healthy BMI. In Australia, there is no GST on basic, healthy foods but 10% GST on discretionary foods, which contributed to the healthy diet being cheaper than the current diet.

The Otago Food Cost Survey [38] collects the price of a diet that meets the NZ Eating and Activity Guidelines and contains some less healthy snack foods but no alcohol or takeaways. The cost of three diets is calculated: basic (cheapest), moderate and liberal (most expensive, most variety). For a similar household of four, the costs of the healthy diets for Māori ($559) and Pacific ($527) in this study were between the cost of the basic ($482) and moderate ($628) diets in the Otago study, while the cost for the total population ($649) was slightly higher than the moderate diet.

There is no accepted benchmark for affordability of a healthy diet internationally, though other researchers consider a household is suffering from food stress if more than 25% of disposable income is spent on food [39]. Therefore, NZ households receiving the minimum wage or income support would be suffering from food stress with some households requiring half of their income to purchase a healthy diet. The percentage of household income required for other major costs such as housing and utilities also determines the income available for food. Affordability was similar for the healthy and current diets for Māori. The healthy diet was slightly more affordable for Pacific and the total population. However, for a household of four receiving income support or minimum wage, a considerable portion of household income is required to purchase either diet. Food insecurity is a concern with 7.3% of NZ households classified as having low food security in the 2008/09 national nutrition survey [2]. In NZ, all foods have 15% GST added [40]. If GST was removed from basic healthy foods, this would improve affordability more for the healthy diet than the current diet.

### 4.1. Strengths

Few reported studies have compared the cost of a hypothetical healthy diet and a current diet, particularly for different population groups. The current diet is based on the common foods reported by the population in a national nutrition survey. The healthy diet is developed to meet food-based dietary guidelines and Nutrient Reference Values. The energy requirement for the current diet reflected the actual BMI of the population rather than using the mean reported energy intake in the survey, which is always under-reported [41]. Takeaway foods and alcohol were included in the current diet as these are common. Overall, the healthy diet met more of the micronutrient recommendations than the current diet though the diet for the adult female (total population only) met the RDI for iron on the current diet but not the healthy diet. This study demonstrated that an expert panel is a useful method for gaining cultural input into the commonly consumed foods, dietary patterns and selection of retail outlets used by Māori and Pacific households. As the national nutrition surveys were not recent, the expert panels offered an up-to-date view on commonly consumed foods.

### 4.2. Limitations

Arbitrary decision points occur at all stages of the process from selecting common foods, selecting items to represent other foods, the amount of each food in the diets, the energy requirement, the definition of a healthy diet, sampling retail outlets and the price selected. The nutrient intake of the current diet was based on older nutrition surveys (2008/09, 2002/03) so may not reflect the nutrient intake of the current diet, however no other data were available.

There is a range of healthy menus that could fit the food-based dietary guidelines and recommended dietary intakes. Only one healthy and one current diet was developed for each population group, so this may not be representative of the average cost if a range of diets were priced. The healthy diet was modelled to be aspirational but when limited discretionary foods were added the cost was similar.

There are other inputs to the cost of producing a household meal, aside from food prices, which could underestimate diet cost, particularly for healthy diets, which may require more preparation. Inputs include time, cooking fuel, transport for groceries, storage, preparation, cooking utensils, cooking space and skills [10].

The cost of the diets was calculated using food prices collected at supermarkets, rather than actual household expenditure that may take into account brand loyalty or purchases from multiple stores. The prices for the different population groups were collected at different times and seasons: Pacific in September 2016, total population in November 2016, Māori in July 2017. The Food Price Index indicated that the price of foods increased by 3.0% from July 2016 to July 2017, particularly fruit and vegetables (8.2%) [42]. Therefore, the relative difference between the healthy and current diets of the different population groups was compared, not the absolute amount. The higher price of fruit and vegetables could be a factor in explaining why the Māori healthy and current diets were a similar price, rather than the healthy diet being slightly cheaper for the Pacific and total population diets. Seasons affect fruit and vegetable prices with fresh fruit and vegetables more expensive in July and September, and close to the average monthly price in November [42], therefore it is expected that the cost differences between the healthy and current diets would persist in seasons where prices are lower.

### 4.3. Implications

A diet modelled on dietary guidelines is not more expensive than the current diet when the reference household also shifts from the estimated current energy intake to the recommended energy intake. This is particularly important for those on low incomes because food costs are typically between a quarter and a half of household budgets indicating they are suffering from food stress. There is a perception that healthy diets are more expensive than those currently consumed [43,44]. However, this research and similar research in Australia [37] indicates it is possible to shift to a healthy diet (that does not exceed energy requirements) from the current, healthy diet without additional cost. Price is only one barrier to healthy eating. Other key influences are taste, traditions, convenience, knowledge and cooking skills [43]. Gathering and gifting food is important in reducing diet costs.

This paper describes the collection of the baseline data. After further price collections, it can be seen whether the healthy diet is increasing in cost at a different rate than the current diet. An analysis of foods in the NZ Food Price Index [45] over ten years indicates the price of healthy foods rose at a similar rate compared to unhealthy foods.

It is recommended that work be conducted with the expert panels on how to translate these findings into a practical health promotion tool for Pacific, Māori and low-income households. Monitoring the price and affordability of diets provides robust data and benchmarks to inform economic and fiscal policies [10]. As demonstrated in this study, having information on the prices of the current and healthy diets is invaluable to demonstrating the impact taxes and subsidies will have on diets.

## 5. Conclusions

Expert panels were invaluable in guiding development of the diets to be costed for specific population groups. In NZ, a lower-energy healthy diet is not necessarily more expensive than the current diet, but discretionary foods make up 36–41% of food costs in the current diet. Strategies to switch current spending on discretionary food and takeaways to healthy food need not cost more. However, overall food security is of concern as a considerable portion of income is required to purchase either a healthy or the current diet in NZ, especially for households receiving minimum wage or income support. In order to consume a healthy diet, policies are required to lower the cost of healthy food or ensure that households have sufficient income after fixed expenses to purchase nutritious, acceptable and safe food.

## Figures and Tables

**Table 1:** Percentage of diet cost of each food group.

Food Group	Healthy Diet	Current Diet	Healthy Diet	Current Diet	Healthy Diet	Current Diet
	All	All	Māori	Māori	Pacific	Pacific
Fruits	18.1%	9.4%	13.6%	8.0%	14.1%	7.0%
Vegetables	17.6%	11.8%	20.8%	10.4%	25.2%	12.9%
Grains	13.8%	6.9%	14.0%	5.5%	15.4%	6.8%
Dairy	11.0%	5.5%	11.2%	6.8%	12.4%	5.2%
Protein	37.9%	31.4%	37.9%	22.0%	30.1%	26.6%
Fats and Oils	1.5%	1.3%	0.7%	1.4%	1.50%	0.9%
Sauces and Spreads	0	2.0%	1.6%	2.3%	0	3.0%
Snacks, sweets	0	6.9%	0	11.5%	0	8.0%
Processed meats	0	4.4%	0	5.3%	0	5.2%
Beverages	0	3.3%	0	5.2%	0	3.8%
Takeaway	0	10.8%	0	15.9%	0	14.9%
Alcohol	0	6.4%	0	5.7%	0	5.8%
Proportion less healthy food	0	35.5%	1.6%	45.9%	0	40.7%
Total cost	$649.06	$671.69	$558.50	$561.68	$526.92	$550.52

**Table 2:** Percentage of household income required to purchase diets.

	Standard Diet		GST off Core Foods	
	Healthy Diet % Income	Current Diet % Income	Healthy Diet % Income	Current Diet % Income
Median Household income ($1733 per week)				
Total population	18.7%	19.4%	16.3%	17.7%
Māori	16.1%	16.2%	14.0%	15.1%
Pacific	15.2%	15.9%	13.2%	15.2%
Minimum Wage ($1115 per week)				
Total population	32.8%	33.9%	28.5%	31.0%
Māori	28.2%	28.3%	24.5%	26.3%
Pacific	26.6%	27.8%	23.1%	26.6%
Income support ($636 per week)				
Total population	51.0%	52.8%	44.4%	48.2%
Māori	43.9%	44.2%	38.2%	41.0%
Pacific	41.4%	43.3%	36.0%	41.4%

## Supplementary Materials

The following are available online at http://www.mdpi.com/1660-4601/15/6/1255/s1. Table S1: Common foods added and removed from diets by Māori and Pacific expert panels, Table S2: Individual and household energy requirements for each population group, Table S3: Number of serves of each food group per week for each household member for healthy and current diets, Table S4: Nutrient intake of household members for healthy and current diets for each population group, Table S5: Edible amount of each common food in the current diet per fortnight for each population group, Table S6: Edible amount of each common food in the healthy diet per fortnight for each population group.
