# Supplementary material for: Cost and Affordability of Diets Modelled on Current Eating Patterns and on Dietary Guidelines, for New Zealand Total Population, Māori and Pacific Households
Source: Int J Environ Res Public Health. 2018 Jun 13;15(6):1255. doi: 10.3390/ijerph15061255 (PMC6025104; doi:10.3390/ijerph15061255)
Supplement: Supplementary file 1 [file ijerph-15-01255-s001.zip › Supplementary Tables.docx]

# Supplementary Tables

**Supplementary Table 1: Common foods added and removed from diets by Māori and Pacific expert panels**

|  | **Foods** |  |  |
| --- | --- | --- | --- |
| Foods removed by Māori expert panel | Grapes  Nectarines  Low-fat milk  Yoghurt – natural, low-fat  Cottage cheese | Wholegrain crackers  Wholemeal pasta  Brown rice  Almonds  Olive oil | Muffin  Plain biscuit  Diet cola  Orange juice |
| Foods added by Māori expert panel | Bok choy  Puha  Watercress  Cheese slices  Cream  Reduced fat milk | Bread rolls, white  Rice crackers  Mussels  Pork chop  Tea  Coffee | Coco pops  Donut  Marmite  Meal flavour sachet  Soy sauce  Sweet chilli sauce  KFC chicken and chips  Energy drink  Vodka |
| Foods not in season so not included in price collection for Māori | Avocado  Feijoa  Plums  Strawberries  Tomatoes | Kamokamo  Sweetcorn |  |
| Foods initially added by Māori expert panel but not in final list | Asparagus – limited season so could only be priced 2 months a year  Rewena bread – usually for special meals  Dripping – not purchased as collected from meat at home  RTDs (alcopops) – already 3 types of alcohol  Nutella – already have two spreads  Garlic bread – already sufficient takeaways | | |
| Foods removed by Pacific expert panel | Nectarines  Sultanas  Capsicum  Courgette  Mushrooms  Cottage cheese  Low-fat milk | Almonds  Chicken breast  Fresh fish fillets  Hummus | Chocolate biscuit  Muffin  Crackers |
| Foods added by Pacific expert panel | Cassava  Garlic  Green banana  Taro  Taro leaves  Tea  Coffee  Reduced fat milk | Cabin bread  Vermicelli  Canned corned beef  Fresh fish, whole  Mutton flaps | Coconut cream bun  Donut  Pineapple pie  Coconut cream  Marmite  Soy sauce  Chow mein  Sushi  Energy drink |
| Foods initially added by Pacific expert panel but not in final list | Ginger – only small amount and represented by garlic  Mango – sufficient fruit  Pineapple canned – represented by canned peaches  Pineapple, fresh – sufficient fruit  Spinach – represented by silver-beet  Panikeke (pancakes) – represented by similar items  Sago  Chicken carcass – too difficult to calculate edible amount  Pigs head, pigs trotters – too difficult to calculate edible amount, low consumption national nutrition survey  Condensed milk – represented by coconut cream  Vegetable oil – represented by canola oil  Lard – low consumption compared to other fats and oils in national nutrition survey  Nutrigrain – sufficient sugary breakfast cereals  Sweet chilli sauce – sufficient sauces  Koko Samoa – represented by milo  Flavoured milk – low consumption national nutrition survey  Sausage roll – represented by meat pie  Deep fried sausage – sufficient takeaway items  RTDs (alcopops) – beer and wine sufficient | | |
| Foods not in season so not included in price collection for Pacific | Feijoa  Watermelon  Capsicum | | |

**Supplementary Table 2: Individual and household energy requirements for each population group**

|  | **Total population** | | **Māori** | | **Pacific** | |
| --- | --- | --- | --- | --- | --- | --- |
| Member of reference household | Target/ current BMI/kg | Energy require-ment MJ | Target/current BMI | Energy require-ment MJ | Target/current BMI/  kg | Energy require-ment MJ |
| ***Healthy diet*** | | | | | | |
| 45-year man | 23.0 | 11.3 | 23.0 | 11.3 | 23.0 | 11.1 |
| 45-year woman | 23.0 | 8.9 | 23.0 | 8.9 | 23.0 | 8.8 |
| 14-year boy | 19.2 | 13.0 | 19.2 | 13.0 | 19.2 | 13.2 |
| 7-year girl | 15.4 | 6.7 | 15.4 | 6.7 | 15.4 | 6.8 |
| Household |  | 39.9 |  | 39.9 |  | 39.9 |
| ***Current diet*** | | | | | | |
| 45-year man | 29.5 | 12.5 | 32.1 | 12.9 | 34.2 | 13.2 |
| 45-year woman | 28.3 | 9.7 | 31.2 | 10.3 | 35.6 | 10.7 |
| 14-year boy | 63.9kg | 14.2 | 69.1kg | 15.0 | 71.6kg | 15.3 |
| 7-year girl | 27.1kg | 7.3 | 30.1kg | 8.0 | 30.9kg | 8.1 |
| Household |  | 43.6 |  | 46.0 |  | 47.3 |

**Supplementary Table 3: Number of serves of each food group per week for each household member for healthy and current diets**

| **Population Group** | **Diet** | **Fruit** | **Vegetables** | **Dairy** | **Grains** | **Meat, seafood, eggs, nuts, legumes** |
| --- | --- | --- | --- | --- | --- | --- |
| 7-year girl | | | | | | |
| Total | Healthy | 14 | 22 | 14 | 44 | 12 |
|  | Current | 12 | 17 | 10 | 24 | 5 |
| Māori | Healthy | 15 | 21 | 16 | 27 | 12 |
|  | Current | 10 | 12 | 8 | 25 | 7 |
| Pacific | Healthy | 14 | 22 | 15 | 39 | 13 |
|  | Current | 12 | 18 | 10 | 24 | 6 |
| 14-year boy | | | | | | |
| Total | Healthy | 20 | 39 | 23 | 79 | 27 |
|  | Current | 8 | 22 | 11 | 44 | 14 |
| Māori | Healthy | 18 | 30 | 22 | 56 | 30 |
|  | Current | 9 | 17 | 15 | 43 | 18 |
| Pacific | Healthy | 20 | 41 | 24 | 77 | 24 |
|  | Current | 8 | 22 | 11 | 43 | 16 |
| Adult woman | | | | | | |
| Total | Healthy | 19 | 28 | 16 | 57 | 14 |
|  | Current | 10 | 25 | 9 | 35 | 13 |
| Māori | Healthy | 14 | 28 | 14 | 41 | 20 |
|  | Current | 10 | 21 | 8 | 25 | 15 |
| Pacific | Healthy | 19 | 28 | 16 | 48 | 15 |
|  | Current | 10 | 25 | 9 | 37 | 12 |
| Adult Man | | | | | | |
| Total | Healthy | 18 | 33 | 18 | 67 | 22 |
|  | Current | 8 | 25 | 9 | 42 | 18 |
| Māori | Healthy | 16 | 31 | 14 | 51 | 26 |
|  | Current | 10 | 21 | 8 | 31 | 22 |
| Pacific | Healthy | 19 | 39 | 20 | 59 | 21 |
|  | Current | 11 | 27 | 6 | 43 | 17 |

**Supplementary Table 4: Nutrient intake of household members for healthy and current diets for each population group**

| **Population Group** | **Diet** | **% energy from fat** | **% energy from saturated fat** | **% energy from carbohydrate** | **% energy from protein** | **Sodium mg** |
| --- | --- | --- | --- | --- | --- | --- |
| ***7-year girl*** |  |  |  |  |  |  |
| Total | Healthy | 26 | 7 | 54 | 20 | 1412 |
|  | Current | 30 | 13 | 54 | 15 | 1920 |
| Māori | Healthy | 29 | 9 | 50 | 19 | 1735 |
|  | Current | 34 | 15 | 51 | 14 | 2956 |
| Pacific | Healthy | 27 | 8 | 53 | 19 | 1781 |
|  | Current | 32 | 13 | 53 | 13 | 2023 |
| ***14-year boy*** |  |  |  |  |  |  |
| Total | Healthy | 29 | 7 | 51 | 20 | 2944 |
|  | Current | 33 | 14 | 50 | 17 | 4075 |
| Māori | Healthy | 29 | 8 | 50 | 24 | 3344 |
|  | Current | 34 | 15 | 50 | 15 | 5617 |
| Pacific | Healthy | 31 | 9 | 50 | 18 | 3458 |
|  | Current | 35 | 14 | 49 | 16 | 4424 |
| ***Adult woman*** |  |  |  |  |  |  |
| Total | Healthy | 27 | 6 | 54 | 19 | 1823 |
|  | Current | 32 | 13 | 46 | 17 | 2575 |
| Māori | Healthy | 25 | 7 | 53 | 24 | 2511 |
|  | Current | 35 | 14 | 46 | 15 | 3287 |
| Pacific | Healthy | 29 | 9 | 49 | 16 | 2120 |
|  | Current | 35 | 14 | 52 | 18 | 3034 |
| ***Adult man*** |  |  |  |  |  |  |
| Total | Healthy | 29 | 7 | 51 | 19 | 2215 |
|  | Current | 32 | 12 | 45 | 18 | 3534 |
| Māori | Healthy | 29 | 8 | 48 | 21 | 2853 |
|  | Current | 37 | 15 | 41 | 16 | 4184 |
| Pacific | Healthy | 31 | 9 | 50 | 19 | 2865 |
|  | Current | 33 | 13 | 46 | 16 | 3728 |

**Supplementary Table 5: Edible amount of each common food in the current diet per fortnight for each population group**

| **Current Diet**  **Per fortnight** | **Total population** | **Māori** | **Pacific** |
| --- | --- | --- | --- |
|  | Edible amount (g) | Edible amount (g) | Edible amount (g) |
| **Fruit** |  |  |  |
| Apples | 1520 | 1190 | 1770 |
| Bananas | 1910 | 2130 | 1980 |
| Grapes | 760 |  | 370 |
| Kiwifruit | 900 | 1039 | 1550 |
| Mandarins | 800 | 1968 | 1295 |
| Nectarines | 600 |  |  |
| Oranges | 1170 | 1162 | 1430 |
| Peaches canned | 660 | 700 | 660 |
| Pears | 710 | 974 | 740 |
| Sultanas | 105 | 70 |  |
| **Vegetables** |  |  |  |
| Avocadoes | 135 |  | 110 |
| Bok choy |  | 290 |  |
| Broccoli | 1015 | 350 | 800 |
| Cabbage | 600 | 770 | 900 |
| Capsicums | 360 | 130 |  |
| Carrot s | 755 | 755 | 500 |
| Cassava, frozen |  |  | 1595 |
| Cauliflower | 550 | 390 | 550 |
| Corn | 180 |  | 450 |
| Courgettes | 400 | 215 |  |
| Cucumbers | 200 | 330 | 155 |
| Garlic |  |  | 7 |
| Green bananas |  |  | 550 |
| Kumara | 1150 | 1370 | 800 |
| Lettuce | 685 | 270 | 600 |
| Mixed vegetables, frozen | 1040 | 1120 | 1505 |
| Mushrooms | 410 | 310 |  |
| Onions | 420 | 790 | 860 |
| Oven baked fries | 1220 | 1040 | 850 |
| Peas green frozen | 950 | 700 | 675 |
| Potatoes | 3880 | 2430 | 3130 |
| Puha |  | 105 |  |
| Pumpkin | 550 | 1140 | 375 |
| Silverbeet | 550 | 436 | 525 |
| Taro |  |  | 1900 |
| Taro leaves |  |  | 105 |
| Tomatoes | 730 |  | 870 |
| Tomatoes canned | 495 | 330 | 235 |
| Watercress |  | 131 |  |
| **Dairy** |  |  |  |
| Cheese Colby | 750 | 760 | 760 |
| Cheese Edam | 300 | 20 | 280 |
| Cheese slices, processed |  | 232 |  |
| Cream |  | 450 |  |
| Milk, low fat | 3610 |  |  |
| Milk, reduced fat |  | 250 | 2650 |
| Milk, whole | 6630 | 11157 | 6380 |
| Yoghurt, flavoured | 1650 | 1170 | 1700 |
| Yoghurt, natural low fat | 150 |  |  |
| **Grains** |  |  |  |
| Bread multigrain | 1880 | 1750 | 1670 |
| Bread rolls, white |  | 600 |  |
| Bread white | 4120 | 5225 | 5630 |
| Bread wholemeal | 960 | 838 | 1300 |
| Pita bread white | 680 |  |  |
| Cabin bread |  |  | 760 |
| Cornflakes | 760 | 180 | 680 |
| Muesli | 330 | 110 | 170 |
| Rice bubbles | 120 |  |  |
| Porridge | 1700 | 530 | 1400 |
| Wheat biscuits | 1045 | 787 | 1095 |
| Pasta | 3270 | 1520 | 1000 |
| Rice white | 2930 | 1210 | 3057 |
| Spaghetti canned | 920 | 1050 | 920 |
| Vermicelli |  |  | 210 |
| **Meat and alternatives** |  |  |  |
| Baked beans | 1020 | 1160 | 1020 |
| Almonds | 120 |  |  |
| Peanuts |  | 210 |  |
| Peanut butter | 285 | 185 | 165 |
| Beef blade steak | 560 | 410 | 690 |
| Beef, corned fresh | 310 | 430 | 360 |
| Beef mince | 1130 | 1070 | 680 |
| Beef rump steak | 570 |  | 435 |
| Chicken breast | 660 | 180 |  |
| Chicken drumstick | 630 | 590 | 680 |
| Chicken rotisserie | 1220 | 510 | 730 |
| Eggs | 945 | 1097 | 1115 |
| Fish frozen fillets | 365 | 415 | 610 |
| Fish, fresh fillets | 365 | 380 |  |
| Fish, fresh whole |  |  | 610 |
| Lamb chop | 520 | 580 | 645 |
| Mussels |  | 660 |  |
| Mutton flaps |  |  | 570 |
| Pork chop |  | 290 |  |
| Pork roast | 560 | 650 | 635 |
| Tuna canned | 360 | 675 | 500 |
| **Fats and Oils** |  |  |  |
| Butter | 435 | 550 | 420 |
| Canola oil | 165 | 564 | 190 |
| Margarine | 490 | 295 | 580 |
| Olive oil | 170 |  | 195 |
| **Discretionary items** |  |  |  |
| ***Snacks, sweets*** |  |  |  |
| 2 minute noodles | 2350 | 1910 | 1200 |
| Biscuit, chocolate | 360 | 719 |  |
| Biscuit, plain | 490 |  | 660 |
| Cake | 325 | 1500 | 345 |
| Chocolate bar | 520 | 560 | 620 |
| Coconut cream bun (panipopo) |  |  | 400 |
| Coco-pops |  | 360 | 240 |
| Crackers Shapes (high fat, high sodium) | 410 | 702 |  |
| Donut |  | 100 | 430 |
| Ice cream | 970 | 1760 | 1170 |
| Lollies | 80 | 245 | 180 |
| Muesli bar | 310 | 784 | 195 |
| Muffin | 480 |  |  |
| Pineapple pie (paifala) |  |  | 470 |
| Potato crisps | 830 | 1380 | 900 |
| Salted peanuts | 230 |  | 360 |
| ***Processed Meats*** |  |  |  |
| Bacon | 170 | 744 | 190 |
| Corned beef canned |  |  | 660 |
| Ham | 380 | 500 | 380 |
| Luncheon meat | 70 | 226 | 80 |
| Sausages | 1390 | 1278 | 1410 |
| ***Sauces and Spreads*** |  |  |  |
| Coconut cream, regular |  |  | 603 |
| Jam | 270 | 30 | 325 |
| Marmite |  | 67 | 120 |
| Mayonnaise | 270 | 180 | 350 |
| Meal flavour sachet (powder) |  | 67 |  |
| Pasta sauce | 470 | 980 | 230 |
| Soup, vegetable canned | 1250 | 517 | 1250 |
| Soy sauce |  | 70 | 70 |
| Sweet chilli sauce |  | 177 |  |
| Tomato sauce | 235 | 486 | 235 |
| White sugar | 515 | 225 | 652 |
| **Takeaways** |  |  |  |
| Burger | 840 |  | 715 |
| Chips | 1040 | 540 | 1070 |
| Chow mein |  |  | 1360 |
| Fish battered | 620 | 670 | 590 |
| KFC chicken and chips |  | 284 | 230 |
| McDonalds combo (burger and chips) |  | 2216 |  |
| Pie meat | 1230 | 940 | 1070 |
| Pizza Hawaiian | 1240 | 170 | 900 |
| Sushi |  |  | 285 |
| **Beverages** |  |  |  |
| Coffee (powder) |  | 14 | 22 |
| Cola | 6900 | 4008 | 6200 |
| Diet cola | 950 |  | 1050 |
| Energy drink |  | 3050 | 550 |
| Fruit drink | 2235 | 3590 | 1985 |
| Milo | 420 | 105 | 420 |
| Orange juice | 2450 |  | 2250 |
| Powdered drink | 25 | 90 | 75 |
| Tea (leaves) |  | 14 | 26 |
| **Alcohol** |  |  |  |
| Beer | 5290 | 3300 | 6160 |
| Vodka |  | 289 |  |
| Wine | 1870 | 800 | 770 |

**Supplementary Table 6: Edible amount of each common food in the healthy diet per fortnight for each population group**

| **Healthy Diet Per fortnight** | **Total population** | **Māori** | **Pacific** |
| --- | --- | --- | --- |
|  | Edible amount(g) | Edible amount(g) | Edible amount(g) |
| **Fruit** |  |  |  |
| Apples | 3380 | 1820 | 3340 |
| Bananas | 3450 | 3680 | 3450 |
| Grapes | 920 |  | 400 |
| Kiwifruit | 1530 | 1900 | 2420 |
| Mandarins | 1480 | 3956 | 1600 |
| Nectarines | 960 |  |  |
| Oranges | 3360 | 1540 | 3360 |
| Peaches canned | 960 | 1284 | 960 |
| Pears | 970 | 1008 | 1770 |
| Sultanas | 320 | 112 |  |
| **Vegetables** |  |  |  |
| Avocados | 700 |  | 800 |
| Bok choy |  | 610 |  |
| Broccoli | 1330 | 1305 | 1250 |
| Cabbage | 900 | 580 | 450 |
| Capsicum | 780 | 540 |  |
| Carrots | 2290 | 2002 | 2660 |
| Cassava, frozen |  |  | 610 |
| Cauliflower | 420 | 620 | 1750 |
| Corn, frozen | 150 |  | 150 |
| Courgettes | 400 | 555 |  |
| Cucumber | 740 | 740 | 740 |
| Garlic |  |  | 28 |
| Green bananas |  |  | 650 |
| Kumara | 1700 | 1550 | 1750 |
| Lettuce | 530 | 439 | 530 |
| Mixed vegetables, frozen | 600 | 735 | 650 |
| Mushrooms | 800 | 910 |  |
| Onion | 410 | 770 | 590 |
| Peas green frozen | 1290 | 420 | 1330 |
| Potato fries, frozen |  | 980 |  |
| Potato | 4300 | 3720 | 1050 |
| Puha |  | 430 |  |
| Pumpkin | 1600 | 1260 | 1000 |
| Silverbeet | 500 | 1265 | 1250 |
| Taro |  |  | 5360 |
| Taro leaves |  |  | 210 |
| Tomatoes | 520 |  | 750 |
| Tomatoes canned | 1030 | 920 | 1490 |
| Watercress |  | 370 |  |
| **Dairy** |  |  |  |
| Cheese Edam | 2000 | 1910 | 2420 |
| Cheese slices, processed |  | 100 |  |
| Cottage cheese | 310 |  |  |
| Milk, low-fat | 10940 |  |  |
| Milk, reduced fat |  | 10128 | 10940 |
| Milk, standard |  | 900 |  |
| Yoghurt, flavoured | 1600 | 5376 | 900 |
| Yoghurt, flavoured reduced fat |  |  | 5890 |
| Yoghurt, natural low fat | 5340 |  |  |
| **Grains** |  |  |  |
| Bread multigrain | 6060 | 6635 | 4560 |
| Bread rolls, white |  | 1388 |  |
| Bread white | 830 |  |  |
| Bread wholemeal | 1800 | 3835 | 6570 |
| Crackers, rice |  | 952 |  |
| Crackers, wholegrain | 1460 |  | 1660 |
| Pita bread white | 1800 |  |  |
| Cornflakes | 150 | 602 | 680 |
| Muesli | 620 | 1345 | 560 |
| Porridge | 4440 | 820 | 2140 |
| Wheat biscuits | 1690 | 1008 | 2030 |
| Pasta | 3600 | 3555 | 1150 |
| Pasta wholemeal | 2100 |  | 2100 |
| Rice brown | 4540 |  | 396 |
| Rice white | 2300 | 2270 | 4300 |
| Spaghetti canned | 600 | 400 | 1000 |
| Vermicelli |  |  | 280 |
| **Meat and alternatives** |  |  |  |
| Baked beans | 1900 | 1500 | 1020 |
| Hummus | 1160 | 715 |  |
| Almonds | 530 |  |  |
| Peanut butter no salt, or sugar | 550 | 555 | 670 |
| Peanuts plain | 420 | 990 | 740 |
| Beef blade steak | 820 | 700 |  |
| Beef, corned |  |  | 920 |
| Beef, corned canned, reduced fat |  |  | 220 |
| Beef mince | 820 | 820 | 920 |
| Beef rump steak |  |  | 840 |
| Chicken breast | 880 | 1430 |  |
| Chicken rotisserie | 860 | 840 | 860 |
| Eggs | 1690 | 1812 | 1810 |
| Fish frozen fillets | 390 | 780 |  |
| Fish, fresh fillets | 530 | 890 |  |
| Fish, fresh whole |  |  | 940 |
| Mussels |  | 746 |  |
| Lamb chop | 460 | 720 | 380 |
| Pork chop |  | 380 |  |
| Pork leg roast | 460 | 660 | 380 |
| Tuna canned | 820 | 1180 | 820 |
| Legumes, canned | 1200 | 840 | 1070 |
| **Fats and Oils** |  |  |  |
| Canola oil | 440 | 705 | 400 |
| Margarine | 660 | 515 | 640 |
| Olive oil | 450 |  | 430 |
| **Other** |  |  |  |
| Coconut cream, lite |  |  | 1131 |
| Marmite |  | 180 |  |
| Pasta sauce |  | 1550 |  |
| Soy sauce, reduced salt |  |  | 104 |
| Vegetable soup, canned |  | 1800 |  |
| **Beverages** |  |  |  |
| Coffee (powder) |  | 12 | 19 |
| Tea (leaves) |  | 8 | 32 |

**Sample menus available on request**
